# Dr. J. H. Benton

**Published:** 1904-11

**Authors:** B. F. Arrington


### DR. J. H. BENTON.

Dr. J. H. Benton, of Newbern, died suddenly at Black Mountain Station, Thursday, September 15, 1904. His remains passed here *en route* for Kinston Saturday morning. Burial service and interment took place Saturday afternoon. He was placed to rest in the family burial lot beside a son and daughter that had preceded him.

Dr. Benton had been in feeble health for several months, and had recently gone to the mountain section to recuperate, but gradually grew worse, and the end came suddenly.

He anticipated the approach of death with composure and had no fears to disturb and harass a peaceful state of mind. He died as he lived,—true in the faith of the love and mercy of a blessed Redeemer, and at peace with all men. Tidings of his death came as a shock to many, and has brought sorrow and sadness to the hearts of a bereaved wife and many loving kindred and friends.

Dr. Benton was truly a good and true man in the fullest acceptation of the term, and discharged well and faithfully the varied duties of life. He was a man of sound judgment, and always acted in strict accord with his honest convictions of what was right, and lived more for others than for self. He was a devoted, loving husband and father, and as a friend there was none truer; and he was ever benevolent, kind, and helpful to the poor and needy. To appreciate his character rightly and to realize fully the beauty of the many noble traits and virtues with which he was so richly endowed, was to know him well. Those who knew him best loved him most.

He had been a successful practitioner of medicine in his native county—Sampson—for more than twenty-five years, and for ten years past had made Newbern his home and had enjoyed a lucrative dental practice, and was held in the highest esteem by all who knew him professionally and personally. He occupied a prominent position as a dentist in the State and in the State Dental Society, and it will be difficult to fill his place. He served well while he lived, and is at rest.

Peace be with him.

B. F. Arrington.

